# Regenerative Surgical Management of an Endodontic Periodontic Lesion of the Mandibular Molar Combined With External Inflammation Root Resorption

**DOI:** 10.1155/2024/1048933

**Published:** 2024-08-29

**Authors:** Henytaria Fajrianti, Fauziah Karimah, Safitri Kusuma Dewi, Diatri Nari Ratih, Nungky Devitaningtyas, Vincensia Maria Karina, Silviana Farrah Diba

**Affiliations:** ^1^ Department of Conservative Dentistry Faculty of Dentistry Universitas Gadjah Mada, Yogyakarta, Indonesia; ^2^ Department of Periodontics Faculty of Dentistry Brawijaya University, Malang, Indonesia; ^3^ Department of Periodontics Faculty of Dentistry Universitas Gadjah Mada, Yogyakarta, Indonesia; ^4^ Department of Dentomaxillofacial Radiology Faculty of Dentistry Universitas Gadjah Mada, Yogyakarta, Indonesia

**Keywords:** Biodentine, demineralized freeze-dried bone allograft, endodontics, endo–perio lesion, furcation involvement, periodontology

## Abstract

Endo–perio lesions are lesions involving pulp tissue with periodontal tissue. The bacterial infection of the pulp can spread to the furcation area through the accessory canal, causing damage to the furcation area. Regeneration therapy has good success when performed with flap surgery and is performed in cases of Grades I and II furcation involvement. Demineralized freeze-dried bone allograft (DFDBA) is a regenerating material that has osteoinductive and osteoconductive abilities. It has the advantage of successful treatment of bone defects. Biodentine is an agent used for direct pulp capping, root perforation and furcation repair, and apexification. It can bind and enter the dentinal tubules and create interlocking crystals with dentin. This case report presents the treatment of furcation involvement Grade II originating from endo–perio lesions by using DFDBA and Biodentine as regeneration materials with 6 months of follow-up.

## 1. Introduction

External root resorption (ERR) is a dynamic process that can cause the loss of periodontal ligaments (PDLs), dental hard tissues, and, in advanced stages, even the tooth pulp. This problem typically begins as small cementum lesions within the PDL and may progress to infect the tooth pulp if left untreated [1, 2].

Traumatic injuries, tooth replacement, intentional (auto)replantation, and autogenous tooth transplantation are just a few causes of ERR. Given that ERR is a degenerative lesion that could cause the gradual loss of dental structure and teeth, it needs to be quickly identified, carefully managed, and correctly treated. In accordance with the cause, severity, and participation of the pulpal tissue in the associated resorption, different treatment approaches are used for ERR [3, 4].

A recent study found that regenerative endodontic procedures (REPs) have the potential to be developed as a treatment for ERR. REPs, however, are not considered to be the first line of treatment and need additional clinical research [3]. Quick endodontic treatment, the elimination of microorganisms, and the use of an appropriate calcium silicate–based biomaterial have been demonstrated to be capable of halting and even reversing root resorption in reimplanted and lost teeth [5, 6].

The important connection between the periodontium and endodontium can be explained by the same mesodermal origin of the associated tissues of the periodontium (the precursor of PDL) and dental pulp (the precursor of dental pulp). Therefore, if bacteria, fungi, or viruses attack one tissue, they will also affect the other [7].

Endo–perio lesions can also be caused by iatrogenic factors, such as perforations, excessive instrumentation, trauma, dental malformations, root resorption, inadequate root canal therapy, dentistry chemicals, over- or underobturation, excessive irrigation, coronal leakage, and poor restorations. Bacteria-infected pulp can travel to the periodontium (retrograde periodontitis) through the lateral canals, furcation canal, or apical foramen, and the same is true for the periodontium [8, 9].

Endodontic and periodontal therapies are required for primary periodontal disease with secondary endodontic involvement and real combination endodontic–periodontal disorders. In these situations, the prognosis heavily depends on the level and response to periodontal therapy for periodontal disease. In the presence of significant bone loss, conventional endodontic and periodontal therapies may be insufficient to maintain the tooth. Therefore, curative and regenerative therapeutic options should be taken into account. Today, many different ways to treat intrabony defects exist. They include guided tissue regeneration (GTR) with barrier membranes, several kinds of grafting materials or their combinations, enamel matrix proteins, and autologous platelet concentrates [9, 10].

The biological foundation of the GTR procedure is to use a barrier membrane to stop the growth of the apical epithelium into the area above the exposed root surface so that PDL cells and osteoblasts can create PDL tissue and alveolar bone. A bone graft is a substance that is inserted between or around fractured or misshapen bones. The primary roles of all bone graft agents are osteoconduction, osteoinduction, and osteogenesis. The graft serves as a pattern or net for osteoconduction that directs the production of new bone. By contrast, the graft stimulates the development of new bone (osteoinduction), while the graft cell itself creates new bone (osteogenesis) [11, 12].

Over the past 30 years, great attention has been paid to the use of demineralized freeze-dried bone allograft (DFDBA), a bone replacement graft that can encourage the regeneration of the attachment apparatus. Ideally, a bone replacement graft should have the ability to initiate osteogenesis. The number of bone morphogenetic proteins that remain after demineralization determines the osteoinductive potential of DFDBA, which is the stimulation of bone formation in extraskeletal regions [13, 14].

## 2. Case Report

### 2.1. Diagnosis and Etiology

**Figure 1:**
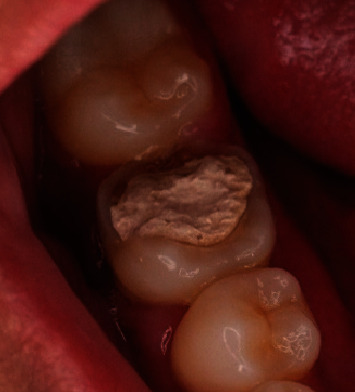
Clinical examination of Tooth #46.

A 29-year-old male patient with a temporary filling of the Mandibular Right Tooth #46 (Figure 1) was referred to the Conservative Department of Prof. Soedomo Dental Hospital, Yogyakarta, Indonesia, by a general dentist.

**Figure 2:**
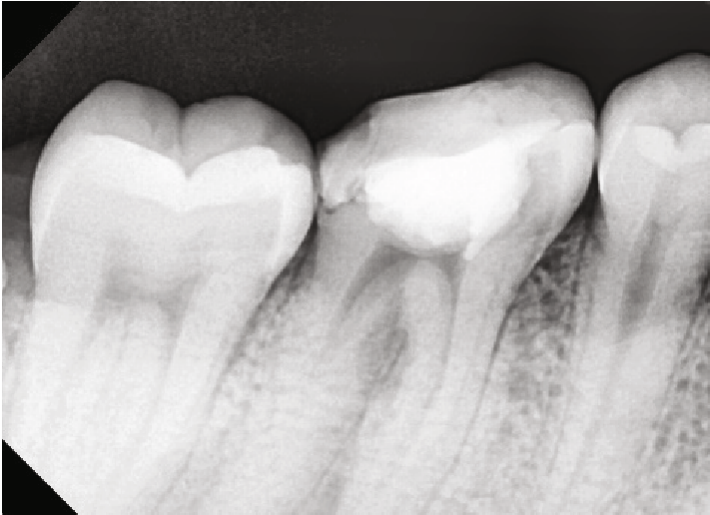
Preoperative radiograph.

Clinical examination indicated a fistule on the buccal gingiva with a probing depth of 6 mm in the buccal area and signs, including spontaneous pain and a positive result on percussion. The #46 tooth has been previously filled, and the patient has a habit of chewing on the right side. Medical history had no influence. Additionally, no substantial family history was disclosed, and the extraoral findings were normal. A radiographic examination of the furcal region of 46 showed a radiolucent area and external resorption (Figure 2). Pulp necrosis, symptomatic apicalis periodontitis, and external resorption were found in Tooth #46.

### 2.2. Treatment Objectives

The chosen procedure was surgical root canal therapy with endo–perio lesions.

### 2.3. Treatment Alternatives

Another treatment choice was considered for this patient. It was a nonsurgical root canal treatment for endo–perio lesions.

### 2.4. Treatment Progress

**Figure 3:**
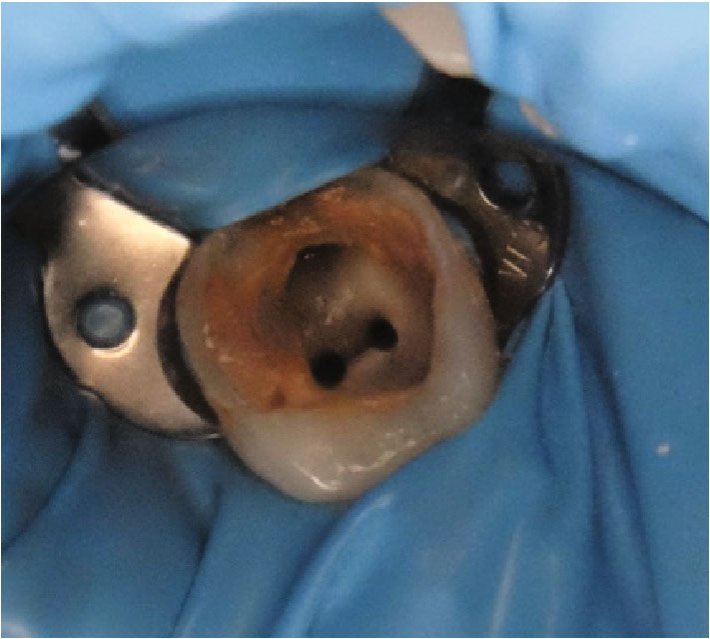
Root canal treatment under rubber dam isolation.

The patient provided his consent. During the first visit, the access cavity was prepared, and initial negotiation was done with a No. 15 stainless steel hand K-file (M-access; Dentsply Maillefer, Ballaigues, Switzerland) under strict rubber dam isolation (Figure 3). A #10 K-file was used to explore the root canals (Dentsply, Switzerland). A SX file was used to perform coronal flaring (ProTaper Gold, Dentsply, Switzerland). The working length was 19 mm for the mesiobuccal, mesiolingual, and distal root canals. Shaping and cleaning were done by using a rotary file (ProTaper Gold, Dentsply Maillefer, Ballaigues, Switzerland).

**Figure 4:**
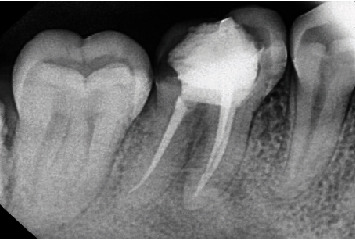
Postoperative radiograph.

Sodium hypochlorite (NaOCl) was used as an irrigant solution during instrumentation, and K-file #25 was used for apical gauging. The tooth was irrigated with a solution of 2.5% NaOCl, 17% EDTA, and 2% chlorhexidine. Obturation was carried out by using epoxy resin (AH Plus, Dentsply), and periapical radiograph evaluation was performed (Figure 4).

**Figure 5:**
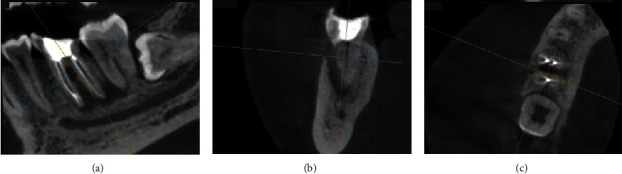
Cone beam computed tomography (CBCT) of Tooth #46 before periodontal surgery. (a) Sagittal view, (b) coronal view, and (c) axial view.

After 3 months of postroot canal treatment, the fistula reappeared in the buccal gingival area. Then, cone beam computed tomography (CBCT) was taken using CBCT (*Vatech*™, Seoul, South Korea), and the images were reconstructed using Ez3D-I software. Periapical lesions that expand distally could still be observed on the sagittal view (Figure 5(a)). External resorption in the furcation area and bone loss at the one-third buccal cervical were revealed (Figures 5(b) and 5(c)). Then, the patient received scaling and root planning, as well as dental health education and probing depth assessment, with a measurement result of 6 mm.

Two weeks later, the control was carried out. Flap debridement surgery and Biodentine application in the ERR area were planned. After the surgical informed consent form was approved, the surgery was performed with the patient's agreement. Local anesthesia was performed.

**Figure 6:**
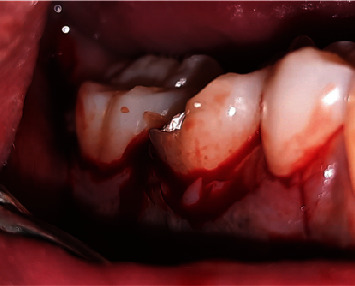
Triangular incision.

**Figure 7:**
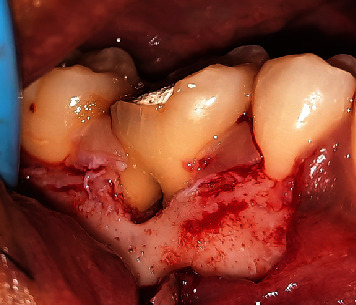
Debridement.

Then, a sulcular incision was produced on Teeth 47 and 45 with a No. 15c blade, a modified Widman flap was executed on Tooth 46, and a vertical incision was performed on Mesial Tooth 45. The interdental papilla area was left alone and deepitelized with a 15c blade to preserve the papilla (Figure 6). A Gracey curette (Osung, South Korea) and an ultrasonic scaler were used to debride and clean the furcation area of Tooth 46 after the full-thickness flap had been reflected (Figure 7).

**Figure 8:**
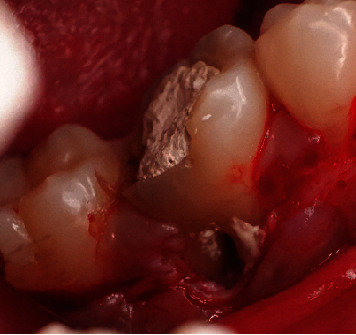
Biodentine application on root resorption.

The granulation tissue was cleaned and irrigated with a sterile saline solution. Subsequently, Biodentine (Septodont, United States) was placed on the resorption tooth's surface and allowed to set for roughly 10 min. Then, the roots were conditioned with EDTA gel and rinsed with saline (Figure 8).

**Figure 9:**
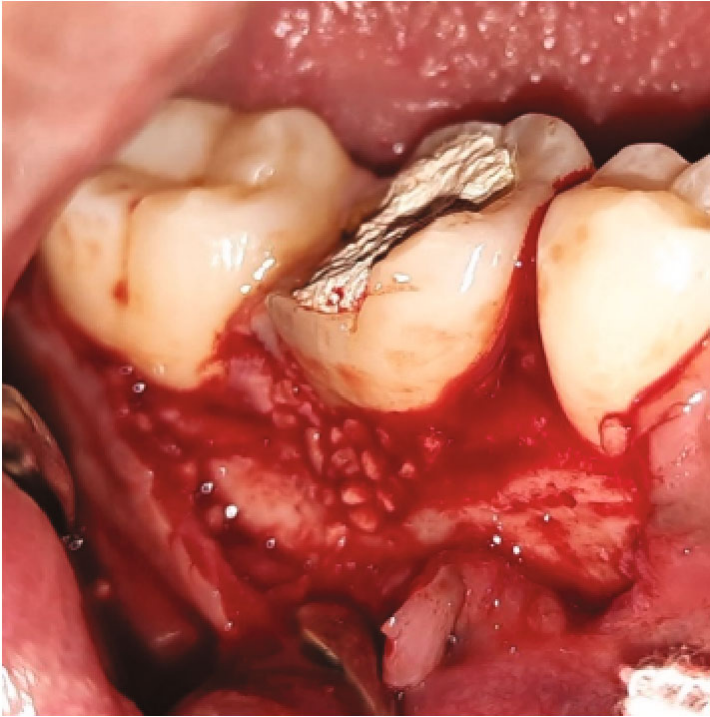
Bone graft and membrane application.

**Figure 10:**
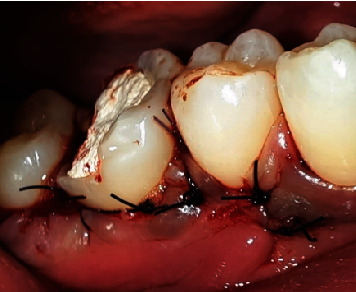
Suturing.

**Figure 11:**
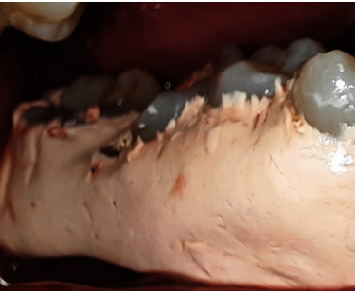
Periodontal dressing application.

The DFDBA bone graft (Batan, Indonesia) was inserted into the area of the bone defect, completely filling the furcation surface, and then coated with a collagen membrane (Collacure, USBIO*®,* China) sewn together with 5.0 monophytic thread (Figure 9). Simple and sling sutures with 4.0 nylon thread were used to seal and stitch the flaps (Figure 10). In addition to giving the patient health instructions, a periodontal dressing (Coe-Pack, GC, Japan) was placed on the surgical area (Figure 11). A prescription for 500 mg each of the painkiller mefenamic acid and the antibiotic amoxicillin was provided to the patient, who was told to take the medication as directed for a week.

**Figure 12:**
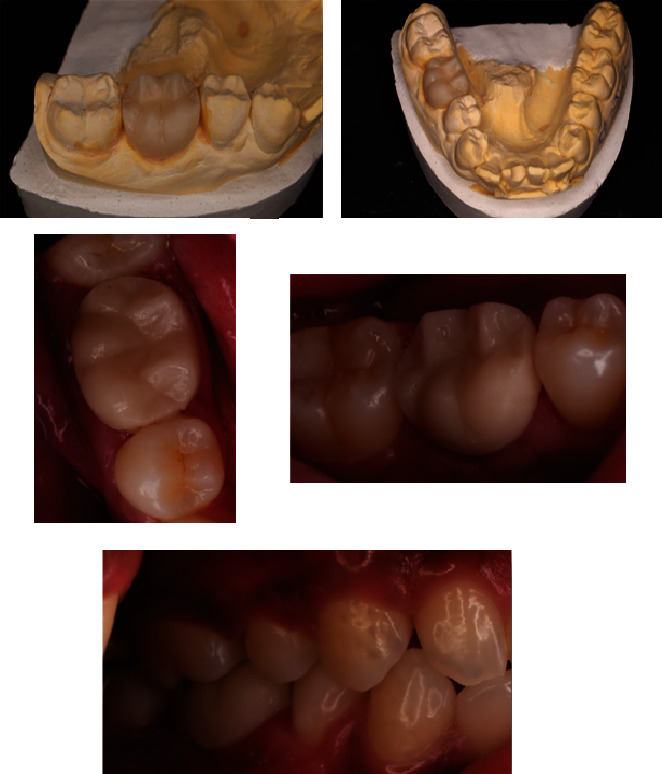
Crown insertion at Tooth #46.

After a week, the patient had no complaints, had completed dental health education, and had the sutures removed. After 1 month, the patient had no complaints, no soft tissue anomalies, and no percussion tenderness. The patient was clinically assessed, and a repeat radiographic examination was performed to assess changes. The examination revealed that the pocket depth had decreased from 6 to 2 mm. The restorative phase began 1 month following the operation. A polysiloxane base (GC, Japan) was used to record the impression. Then, temporization was performed. One week later, composite crowns were bonded to the tooth (Figure 12).

**Figure 13:**
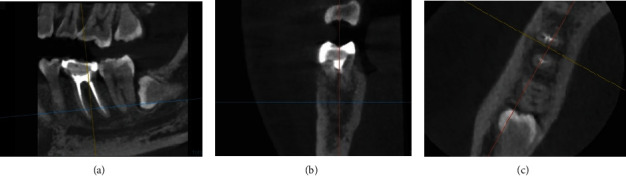
Cone beam computed tomography (CBCT) of Tooth #46 at 3-month follow-up. (a) Sagittal view, (b) coronal view, and (c) axial view.

An inspection revealed that the probing depth was 2 mm during the control period of 3 months. CBCT was done during this period. The periapical and furcation areas showed indications of new bone formation, which was characterised by radiolucent areas that gradually shifted to radiopaque (Figures 13(a), 13(b), and 13(c)).

### 2.5. Treatment Results

**Figure 14:**
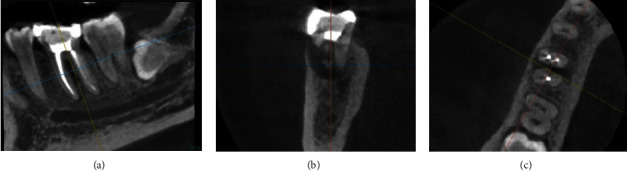
Cone beam computed tomography (CBCT) of Tooth #46 at 6-month follow-up. (a) Sagittal view, (b) coronal view, and (c) axial view.

**Figure 15:**
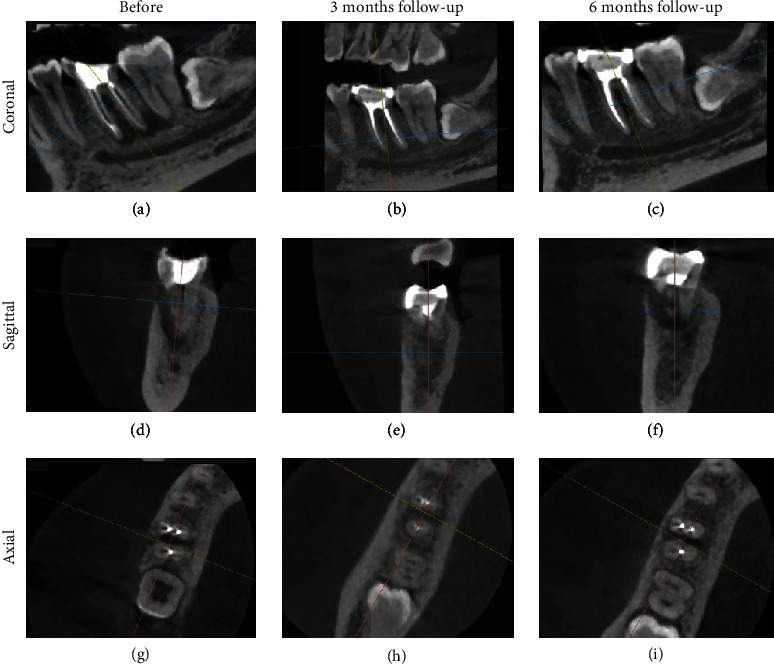
CBCT (a, d, g) before periodontal treatment, (b, e, h) 3 months, and (c, f, i) 6 months of follow-up arranged sequentially, consisting of coronal, sagittal, and axial views.

After 6 months after the surgery, the patient had no complaints and was wearing a crown that was found to be well-suited at the time of the visit. The probing examination was 2 mm. CBCT on the sagittal view showed compacted bone in the periapical area which has a similar appearance to the surrounding normal bone trabecular (Figure 14(a)), while bone defect in the furcation area has remained as seen in the coronal and axial views (Figures 14(b) and 14(c)). Comparison of the condition of Tooth 46 and the periapical status before surgery, after surgery, and after a 6-month follow-up evaluated through CBCT imaging (Figure 15).

## 3. Discussion

Root resorption is the process that removes cementum and/or dentine through the normal or pathological activity of tooth-resorbing cells, which may also be referred to as dentoclasts. Internal and exterior resorptions are the two different categories of tooth resorption. External tooth resorption has been divided into four types on the basis of clinical and histological characteristics: external surface resorption, external inflammatory root resorption, replacement resorption, and ankylosis [4, 5].

Clinicians are challenged by diagnosis in the determination of the treatment plan and long-term prognosis of teeth with endo–perio lesions. One of the most frequent concerns in current clinical practice is the treatment of complex endodontic periodontal disease. Involved teeth might have a worse prognosis if pulpal and periodontal tissues are simultaneously damaged [12, 15].

Furthermore, understanding the etiological component that generates a clinical disease correctly is important for the effective management of the disease. In the present case, severe damage was the key contributing element of resorption. The resorptive abnormalities were the end outcome of a chronic low-grade infection that developed because the impacted tooth was untreated for a prolonged period of time. Injury from trauma and the inflammation of the periodontium and tooth pulp created the lesions, which were then triggered by the damage. After an infection takes hold in the root canal space, bacteria and tissue damage by-products may cause the surrounding periodontal tissue to inflame, which might induce the gradual resorption of the root due to inflammation [15, 16].

In the present case, orthodontic treatment in the past and chewing on one side frequently may have predisposed the patient to EIRR.

The main factors that cause root resorption are inflammation around the surface of the root and the loss or change of the protective layer (precementum or predentin) on the surface of the root. As a result, external resorption in this case was also concentrated in the buccal aspect's middle third likely because the traumatic events that caused direct or indirect harm started the resorptive process [4, 6].

Under such circumstances, successful outcomes can be achieved through early discovery, fault removal, and restoration with a compatible material. Such faults can be repaired with bioactive compounds, such as MTA and Biodentine. Biodentine may be a good choice because it may function as a dentine substitute and be aesthetically acceptable. A regenerative strategy is best because it can restore lost periodontium by creating new attachments and protect against the negative consequences of the disease [5, 6].

The surgical techniques GTR, bicuspidization, tunneling, and root amputation can all be utilized to treat furcation involvement. When combined with a flap debridement operation, regenerative medicine treatment produces positive outcomes [16]. In the present case, flap debridement was done above the right first molar on the mandible, and collagen membrane and DFDBA regeneration material were applied. In the sixth month of control, the probing depth decreased from 6 to 2 mm.

DFDBA's osteoconductive properties enable it to promote bone repair in defects. The bone morphogenic protein (BMP) and growth factors that are generated during acid demineralization confer DFDBA with osteoconductive properties. By encouraging the growth of new blood vessels in the alveolar bone, the BMP can accelerate regeneration. On days 90 and 180 after radiografting, the bone density increased [17]. When the DFDBA bone graft was applied in the third and sixth months, the bone density in the area of the furcation increased.

The radiographic image taken at 6 months revealed an improvement in the resorption of the mesial root in the furcation. The increase in radiodensity on the radiograph taken 9 months after therapy showed that the combination of DFDBA treatment with Biodentine yielded good treatment outcomes. In this study, a CBCT examination was performed prior to surgery, followed by a 3-month and 6-month postoperation in order to observe the changes in new bone formation around the furcation and periapical area. A multiplanar view in CBCT could clearly illustrate the complexity of the furcation area without any superimposition, thus making it reliable for evaluating bone graft treatment [18].

Clinicians face a hurdle when determining the diagnosis and prognosis of a tooth with endo–perio lesions. A proper diagnosis is crucial to define the course of treatment and the long-term prognosis. However, the treatment of a complex endodontic periodontal disease is one of the most frequently encountered difficulties in modern clinical practice. The diagnosis may become increasingly challenging, and, as a result, the prognosis of the implicated teeth may be affected if pulpal and periodontal tissue loss are simultaneously present. This situation emphasizes how crucial following a key diagnostic strategy is to guarantee an appropriate treatment plan and calls for a detailed understanding of the two complicated tissues involved in wound healing [1, 2].

Endodontic treatment and periodontal regeneration therapy are necessary for the treatment of endo–perio lesions. The initial step of the treatment plan is to focus on cleaning and disinfecting the root canal system and is followed by a waiting period. Periodontal surgery aims to eliminate all dead tissue from the surgical site, promote the growth of new hard and soft tissue, and create new attachment structures. Moreover, it could be interesting to test other therapies that recently showed promising results, such as ozonized gels in nonsurgical periodontal treatment [19].

The limitation of the present report is the lack of long-term evaluation until there is more than 1 year of follow-up to come to a generalized conclusion about the success of the treatment. In the first year of follow-up after endodontic treatment and surgical periodontal procedures, bone regeneration is not yet fully visible in the previously defective area, and the lesions appear to be shrinking.

However, the successful periodontal treatment of such lesions has become achievable with the development of novel regeneration materials. In a combined endo–perio lesion, effective endodontic therapy will typically result in the healing of the endodontic component, and the prognosis ultimately depends on the effectiveness of periodontal repair/regeneration initiated by any of the therapeutic modalities [8, 9].

## 4. Conclusion

Early diagnosis and adequate restorative and regenerative material coupled with the appropriate treatment strategy are essential for the long-term and positive prognosis and retention of teeth with ERR and endo–perio lesions.

Regenerating agents, such as DFDBA, also show promise for the treatment of bone defects in endo–perio lesions.

## Data Availability

The data that support the findings of this study are available from the corresponding author, Henytaria Fajrianti, upon reasonable request.
